# An Enhanced Intrusion Detection Model Based on Improved kNN in WSNs

**DOI:** 10.3390/s22041407

**Published:** 2022-02-11

**Authors:** Gaoyuan Liu, Huiqi Zhao, Fang Fan, Gang Liu, Qiang Xu, Shah Nazir

**Affiliations:** 1 College of Intelligent Equipment, Shandong University of Science and Technology, Tai’an 271000, China; liugaoyuan@sdust.edu.cn (G.L.); fangfan@sdust.edu.cn (F.F.); lgtodd@163.com (G.L.); 2 College of Computer Science and Engineering, Shandong University of Science and Technology, Qingdao 266590, China; xuqiang@sdust.edu.cn; 3 Department of Computer Science, University of Swabi, Swabi 23430, Pakistan; snshahnzr@gmail.com

**Keywords:** wireless sensor networks, intrusion

## Abstract

Aiming at the intrusion detection problem of the wireless sensor network (WSN), considering the combined characteristics of the wireless sensor network, we consider setting up a corresponding intrusion detection system on the edge side through edge computing. An intrusion detection system (IDS), as a proactive network security protection technology, provides an effective defense system for the WSN. In this paper, we propose a WSN intelligent intrusion detection model, through the introduction of the k-Nearest Neighbor algorithm (kNN) in machine learning and the introduction of the arithmetic optimization algorithm (AOA) in evolutionary calculation, to form an edge intelligence framework that specifically performs the intrusion detection when the WSN encounters a DoS attack. In order to enhance the accuracy of the model, we use a parallel strategy to enhance the communication between the populations and use the Lévy flight strategy to adjust the optimization. The proposed PL-AOA algorithm performs well in the benchmark function test and effectively guarantees the improvement of the kNN classifier. We use Matlab2018b to conduct simulation experiments based on the WSN-DS data set and our model achieves 99% ACC, with a nearly 10% improvement compared with the original kNN when performing DoS intrusion detection. The experimental results show that the proposed intrusion detection model has good effects and practical application significance.

## 1. Introduction

With the rapid development of real-time big data and the Internet of Things, the demand for surrounding environmental data has also increased significantly [1]. The demand for WSN products with low node costs and easy deployment will gradually expand. WSN products can break through traditional detection methods. They reduce the costs of environmental testing, and also greatly reduce the cumbersome process of traditional testing methods. As a new network, the WSN has been widely studied by scientific researchers and widely used in industry since its inception. Common applications include scenarios such as environmental detection, military operations, and information positioning. In the face of complex and changeable application scenarios, WSN nodes face various challenges, such as (1) the computing power and storage capacity of a single node is quite limited; (2) the communication capability between nodes is weak; (3) the sensor node is in a complex physical environment; (4) some mobile nodes may cause the network topology to be dynamic and random. Therefore, the security of WSN sensors is relatively low, network attacks against WSN sensors are easier, and their security problems are becoming increasingly severe.

Intrusion detection is the second line of defense for network security. An intrusion detection system (IDS) can not only resist network attacks from intruders but also strengthen the system’s defense capabilities based on known attacks. From the perspective of data sources, we can divide intrusion detection systems into three categories: (1) host-based intrusion detection systems [2,3,4], which do not require the participation of network data, and only judge whether the data are abnormal from the intrusion detection library inside the system—this method will waste a lot of CPU resources and it is not suitable for the use of small distributed devices; (2) network-based intrusion detection systems [5,6] can acquire real-time network data packets and establish a corresponding intrusion detection library to perform pattern matching and frequency analysis and judgment on the data packets, but this method will carry large costs for the database update; (3) distributed intrusion detection systems [7,8,9], where the system can comprehensively consider the above two intrusion detection systems, i.e., it can not only detect the operating data of the host but also can detect network data. A WSN, as an excellent distributed device that has a low price, can be widely deployed on the edge side of the Internet of Things to protect the security of the entire Internet of Things system. Intrusion detection is classified by the detection technology, which can be divided into misuse-based intrusion detection [10] and anomaly-based intrusion detection [11,12]. The first method mainly detects data directly based on the existing signature database of the system; a problem occurs when the system’s signature database is not updated in time, and thus the new intrusion behaviors cannot be detected. In the second method, the system will establish a normal working database, and then compare this with the behavior of the computer; if it does not exceed the threshold, it will be a normal behavior; otherwise, the system will activate an alarm. This method is also the most common intrusion detection method, but this method often encounters challenges wherein the non-attack behavior is not within the scope of the normal working mode; the false positive rate of this method is relatively high. However, the application of intrusion detection systems on WSNs presents a great challenge. Because WSNs cannot provide enough information required by traditional intrusion detection technology, traditional intrusion detection technology cannot be directly applied to WSNs. Therefore, building a simple and lightweight WSN intrusion detection technology has become an important issue in the field of WSN security.

Intrusion detection technology is an important technique to ensure product security. Therefore, it is very important to accurately identify various attacks in the network. At present, there are some mature WSN intrusion detection systems based on convolutional neural networks [13], random forest [14], naive Bayes [15], decision tree [16], and other intrusion detection systems based on machine learning. We know that distributed equipment has extremely high requirements regarding space complexity. The space complexity of a convolutional neural network is $S\left(k^{2}\times Cout\times Cin\right)$, where k is the kernel size; the space complexity of random forest is $S\left(N+D\times Split\times TreeNum\right)$, where N is the number of samples and D is the number of features; and the space complexity of naive Bayes is $S\left(N\times D\right)$, but naive Bayes must ensure that the samples are independent of each other to ensure a good effect. kNN [17], with almost the same space complexity as Naive Bayes, is also an excellent machine learning method, whose space complexity is $S\left(N\times D\right)$. The k-Nearest Neighbor (kNN) method is a normal machine learning method, whose structure is simple and easy to implement, and the classification effect is good. Because it eliminates the training process of neural networks, it is often used as a lightweight machine learning method in the intrusion detection systems of traditional networks. Compared with traditional networks, due to the small size of the WSN sensor and low computing power, purely embedding the artificial intelligence model into the WSN sensor may cause slower operation and low performance. Therefore, we can use edge nodes close to the WSN sensor nodes to train and deploy machine learning models at the edge of the network, closer to users and data sources, in an edge-wise manner [18,19,20]. There are two problems when we want to use kNN: one is the measurement of distance, and the other is the selection of the k parameter. For the distance measurement problem, kNN needs to calculate the distance between each sample size of the test set and the training set, which means that it is challenging for kNN to process larger data in reality. Tan et al. [21] used a different strategy to refine the incoming data and greatly improve the classification effect of kNN; for the selection of the k value, Liang [22] and others have made some progress. Huang uses a weighted method to improve kNN. However, these works have not simultaneously optimized the k and weighted above-mentioned problems.

Optimization is one of the most commonly used methods to solve complex problems. For kNN distance selection and K value selection, many scholars also use optimization algorithms to solve them. For example, Chen [23] used the PSO algorithm to optimize kNN weights. Xu [24] optimized the kNN distance formula through GWO [25], and specifically solved the kNN distance selection problem; Tahir [26] et al. optimized the k value selection of kNN through TS [27], and specifically solved the K value selection problem. Of course, common optimization algorithms include evolutionary-inspired algorithms (GA) [28], differential evolution algorithm (DE) [29], whale algorithm (WOA) [30], cat colony algorithm (CA) [31], multiverse algorithm (MVO) [32], and quasi-affine optimization algorithm (QUATER) [33] inspired by natural physics. For WSN nodes with weak computing power, overly complex optimization algorithms cannot be selected for optimization. Therefore, choosing a lightweight and high-performance optimization algorithm is particularly meaningful in the process of WSN intrusion detection. The Arithmetic Optimization Algorithm (AOA) is a new stochastic optimization algorithm proposed by Australian scholar Mirjalili in 2020 [34]. The algorithm has few control parameters, a simple structure, ease of implementation, and has an excellent performance in a variety of industrial optimization problems. In this regard, this article will focus on using the AOA algorithm to optimize the kNN weight and k value selection. However, considering the no free lunch theorem [35], this paper will also compare the kNN optimized by other optimization algorithms with the improved version of the AOA algorithm that we propose.

In this article, we mainly offer the following contributions:

- We propose an intelligent intrusion detection model based on edge intelligence that is deployed at the edge of the WSN node (${\mathrm{kNN}}_{\mathrm{PL}-\mathrm{AOA}}$);
- We propose a parallelized arithmetic optimization algorithm and achieve outstanding results compared to another algorithm;
- Through standard data set testing, our edge intelligent intrusion detection model has good performance in detecting DoS attacks.

The main structure of the article is as follows:

In Section 2, we describe related works to introduce AOA and kNN, which we use in this article. In Section 3, we introduce the improved version of AOA proposed in this article in detail and compare it with other algorithms; Section 4 introduces the intrusion detection system based on WSN, which includes the improved kNN formula and the method of combining PL-AOA and kNN; Section 5 describes the simulation experiment and data statistics of WSN intrusion detection; Section 6 provides the conclusions and future work.

## 2. Related Works

### 2.1. Arithmetic Optimization Algorithm (AOA)

The Arithmetic Optimization Algorithm (AOA) is a new type of swarm intelligence optimization algorithm proposed by Mirjalili in 2020 The algorithm has a simple structure, fewer parameters, and is easy to implement. Its search process is mainly controlled by basic mathematical operators, namely multiplication (M “×”), division (D “÷”), subtraction (S “−”), and addition (A “+”)).

Arithmetic Optimization Algorithms are used to solve optimization problems. First, AOA is achieved by creating multiple initial random candidate solutions $X\in \left(x_{1},x_{2},x_{3},\ldots ,x_{n}\right)$. After the AOA algorithm is initialized, it will first enter the exploration stage. Before the exploration stage, Math Optimizer Accelerated (MOA) is calculated. MOA is obtained by the following Equation (1):

(1) $$MOA\left(C\_iter\right)=Min+C\_iter\times \left(\frac{Max-Min}{M\_Iter}\right)$$

where C_Iter represents the current iteration $C\_Iter\in \left(1,\;M\_Iter\right)$. Max and Min respectively represent the maximum and minimum values of the acceleration function. $MOA\left(C\_Iter\right)$ represents the function value in the tth iteration, which is obtained by Equation (2):

(2) $$x_{ij}\left(C_{Iter}+1\right)=\left\{\begin{array}{c} best\left(x_{j}\right)÷\left(MOP+\epsilon \right)\times \left(\left(ub_{j}-lb_{j}\right)\times \mu +lb_{j}\right),r_{2}<0.5\\ best\left(x_{j}\right)\times MOP\times \left(\left(ub_{j}-lb_{j}\right)\times \mu +lb_{j}\right)\;\;\;\;\;\;\;\;\;\;,r_{2}\geq 0.5 \end{array}\right.$$

where $x_{i}\left(C\_Iter+1\right)\;$ represents the *i*-th solution in the next iteration, $x_{ij}\left(C\_Iter\right)$ represents the j-th position of the *i*-th solution in the current iteration, $best\left(x_{j}\right)$ is the j-th position in the best iteration, ϵ is a small integer number, $ub_{j}$ and $lb_{j}$ represent the upper limit and lower limit of the j-th position, respectively, µ=0.5.

According to the arithmetic operators, using the division (D) operator or the multiplication (M) operator can obtain highly distributed values or decisions. These will help the algorithm exploration mechanism, by using multiplication (M) or division (D). Due to the exploration mechanism of AOA, it is possible to find the approximate optimal solution through multiple iterative explorations in the solution space, thereby providing the possibility to obtain a more promising optimal solution in the subsequent optimization phase (development phase). MOP is a mathematical optimizer; $MOP\left(C\_Iter\right)$ represents the function value of the t-th iteration, as shown in Equations (3) and (4):

(3) $$MO\;P\left(C\_Iter\right)=1-\frac{C\_Iter^{\frac{1}{\alpha }}\;}{M\_Iter^{\frac{1}{\alpha }}}$$

where C_Iter represents the current iteration, and $\left(M\_Iter\right)$ represents the maximum number of iterations. α is a sensitive parameter, which defines the development accuracy in the iterative process; in this paper, α=5.

(4) $$x_{ij}\left(C_{Iter}+1\right)=\left\{\begin{array}{c} best\left(x_{j}\right)+MOP\times \left(\left(ub_{j}-lb_{j}\right)\times \mu +lb_{j}\right)\;\;\;\;\;,r_{3}\geq 0.5\\ best\left(x_{j}\right)-MOP\times \left(\left(ub_{j}-lb_{j}\right)\times \mu +lb_{j}\right)\;\;\;\;\;\;,r_{3}<0.5 \end{array}\right.$$

In order to effectively balance the exploration and development stage of the algorithm, $r_{1}$ is used in AOA as the condition for the algorithm to transition from exploration to development. $r_{1}$ is a random number $\left[0,1\right]$. When $r_{1}>MOA$, the candidate solution tries to approximate the most optimal solution and diverges when the algorithm in the exploratory stage. When $r_{1}\leq MOA$, the candidate solution tends to approximate the optimal solution when the algorithm in the development stage.

### 2.2. K-Nearest Neighbor (kNN)

The K-Nearest Neighbor (kNN) algorithm is a theoretically mature method and one of the most commonly used machine learning algorithms, which is widely used in various practical problems [36,37,38]. The basic idea of the kNN algorithm is that, in the feature space, if most of the k-nearest samples near a sample belong to a certain category, the sample also belongs to this category. In kNN, when a new instance appears, the k-nearest instance is directly found in the training data set, and this new instance is assigned to the class with the largest number of instances among the k training instances. Of course, for kNN, the classification result is affected by three factors: k  value setting, distance measurement method, and decision rules. Among them, decision rules often follow “the minority obeys the majority”. For optimization, the focus is often on optimizing the k value and distance test.

The k value is the only parameter of the kNN algorithm. The selection of the k value has a very important impact on the prediction results of kNN. A small k value will lead to a large prediction error and even noise. A large k value will cause underfitting. Under these circumstances, the forecast model is too simple. For the distance measurement of spatial samples, it is also a crucial factor that affects kNN prediction. Commonly used distance measurement methods include Euclidean distance, Mahalanobis distance, angle cosine distance, etc. In the calculation process, the smaller the distance between the two samples, the closer similarity of the two samples; otherwise, the similarity of the two samples is not good.

Suppose that there are two samples $x_{i}$ and $x_{j}$ in the D dimensional feature; we easily formulate the samples as: $x_{i}=\left(x_{i1},\;x_{i2},\ldots ,\;x_{iD}\right)$ and $x_{j}=\left(x_{j1},\;x_{j2},\ldots ,\;x_{jD}\right)$. The distance between two samples is $d\left(x_{i},\;x_{j}\right)$. Normally, kNN uses Euclidean distance to measure the similarity between samples, as shown in Equation (5):

(5) $$d\left(x_{i},x_{j}\right)=\sqrt{\sum_{k=1}^{D}{\left(x_{ik}-x_{jk}\right)}^{2}}$$

## 3. Improved AOA

In this section, we explain in detail the main ideas and implementation schemes of PL-AOA. We show that it can overcome the problem of AOA, which has slow convergence speed and prematurely falls into the local optimum when solving complex problems.

### 3.1. Lévy AOA

#### Lévy Flight

Compared with Gaussian mutation, Lévy flight [39] random walk is a better search strategy; its flight step size satisfies a stable distribution with heavy tails. By using Lévy flight in the optimization algorithm, we can expand the search range, increase the diversity of the population, and make it easier to jump out of local convergence. The updated formula of Lévy flight is given in Equation (6):

(6) $$x_{i}^{t+1}=x_{i}^{t}+\alpha \times L\overset{´}{e}vy\left(\lambda \right)$$

where $x_{i}^{t}$ represents the position of $x_{i}$ and the t-th generation, × is the point multiplication operation, α is the Levy flight step control amount, $Lévy\left(\lambda \right)$ is the random search path, and Lévy ~ u=t^−λ, Λ∈1,3.

In this section, we effectively combine Lévy flight with the AOA algorithm. We add Lévy flight to the iterative process of the AOA algorithm, which enhances the population diversity of the algorithm, to improve the problem of slow convergence of the AOA algorithm and solve complex problems wherein the system prematurely falls into the problem of local optima. After the AOA algorithm updates the solution, the Lévy flight leads the solution to a new x′ as shown in Equation (7):

(7) $$x_{ij}^{\prime }=x_{ij}+\alpha \times L\overset{´}{e}vy\left(\lambda \right)$$

### 3.2. Parallel Lévy AOA (PL-AOA)

#### Parallel Strategy Based on Lévy AOA

In order to solve this problem, we adopt a parallel strategy to compensate for the negative effects of Lévy flight in order to enhance the communication capabilities between the populations. Normally, this method does not increase the complexity of the algorithm. The population size is taken as 40, divided into four groups [40], and the algorithm steps are as follows:

(1) Initialization:

Initialize the population P and related parameters, and divide P into 4 groups: $g_{i}\;\left(i=1,2,3,4\right)$.

(2) Evaluation:

Evaluate the fitness value of each particle in the population.

(3) Update:

Use Equations (2) and (4) to update the position of each particle. Then, update each group’s historical optimal pbest and global optimal $gbest_{i}\;\left(i=1,2,3,4\right)$.

(4) Communication:

Communicate between groups every k generations: choose the best solution in the group for Lévy flight.

(5) Termination:

Repeat steps 2 to 5. If the predefined function value has been obtained or all iterations have been completed, record the global optimal particle and its fitness value until the end of the optimization process.

**Figure 1:**
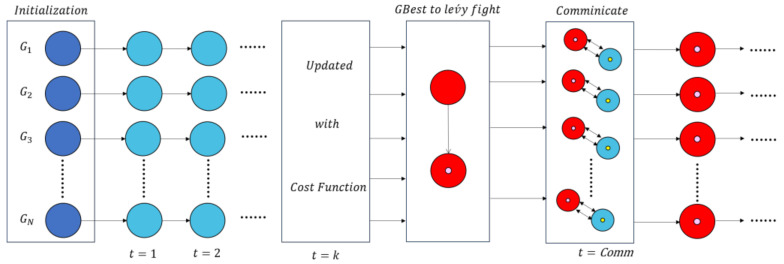
The PL-AOA algorithm flow.

In addition, the flowchart and pseudo code of the algorithm are shown in Figure 1 and Algorithm 1.
**Algorithm 1** Pseudocode of PL-AOA1: Initialize the parameters related to the algorithm: ub,lb,Dim,max_itergroup=4.2: Generate initial population X containing N individuals $X_{i}\left(i=0,1,2,3,\cdots ,N\right)$.3: Divide X into 4 groups.4: Do5:    if $r_{1}>MOA$
6:          Update the X by Equation (1).7:     else8:          Update the X by Equation (2).9:     for i = 1:group
10:          for i = 1:Dim
11:            if $f_{winner}<f_{gbest}$
12:               Update the best solution obtained so far.13:               Change flight status according to iteration.14:            end15:            if iteration=50
16:               Update the pbest by Equation (6) and calculate its fitness value.17:            if $f_{gbest}<f_{pbest\prime }$
18:               Update the best solution obtained so far.19:               Change flight status according to iteration.20:        end21:     end22: While (t<max_iter)$or\;\left(\;get\;the\;expected\;function\;value\right).\;$
23: Return the best solution obtained so far as the global optimum.

## 4. An Edge-Intelligent WSN Intrusion Detection System

### 4.1. Weighted kNN

Though the application of Euclidean distance is more common, it is suitable for situations where the metric standards of each component of the sample vector are uniform. For most statistical problems, since the values of the sample components contribute equally to the Euclidean distance, satisfactory results are often not achieved. Especially when the dimensional difference in the fluctuation range of each component is large, this will cause the contribution of each component to the overall difference to be large, and even the contribution of a certain sample will be almost negligible. When each component is a quantity of a different nature, the size of the Euclidean distance is related to the unit of the sample component. In practical applications, it is impossible to guarantee that the value of a certain dimension has a small fluctuation range, so we can consider weighting $s_{k}\left(k=1,2,\ldots ,D\right)$ for each coordinate sub-scalar. In other words, the coordinates with large changes have smaller weight coefficients than those with small changes, and the differences between different attributes of the samples are quantified to the same interval. This is the method of standardized Euclidean distance, and the formula is as follows in Equation (8):

(8) $$d\left(x_{i},x_{j}\right)=\sqrt{\overset{D}{\underset{k=1}{\sum }}\frac{{\left(x_{ik}-x_{jk}\right)}^{2}}{s_{k}}}$$

As a commonly used machine learning algorithm, many scholars have also improved it, mainly around the adjustment of the k parameter [41,42]. However, no in-depth research has been performed on the determination of *k*, selection of distance functions, or setting of distance weights. For all sample-based distributions, data characteristics, and analysis requirements, they can all be regarded as typical optimization problems. With the help of the optimization ability of a meta-heuristic algorithm, we can obtain a more reasonable and effective kNN classification model.

### 4.2. PL-AOA Combined with kNN

The kNN parameter k and the distance weight $s_{i}$ largely determine the classification effect. However, these two parameters are often considered to be determined under normal circumstances. The PL-AOA proposed in this article can be used to optimize the relevant parameters of kNN to obtain the best or near-best classification results.

**Figure 2:**
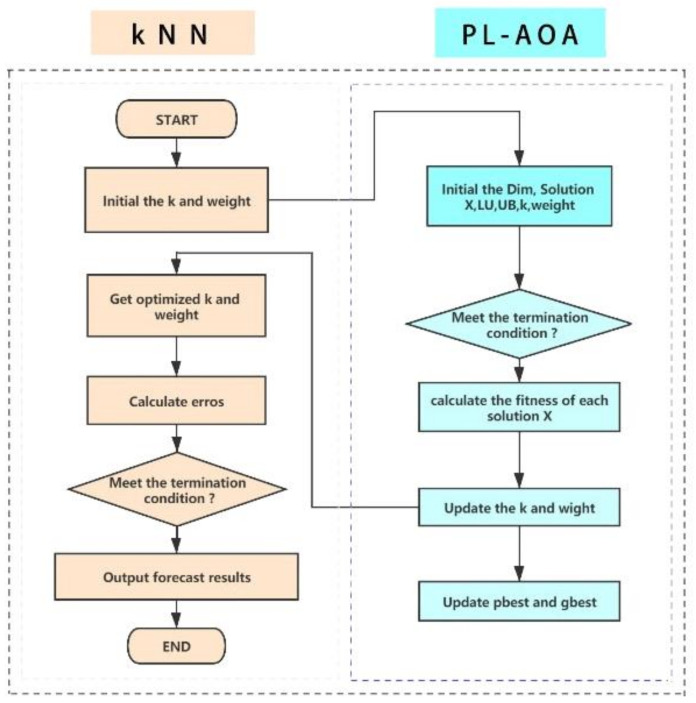
PL-AOA combined with kNN.

The samples in the D  dimensional feature space correspond to the N solution vectors $X_{i}\left(i=1\ldots N\right)$ of the evolutionary algorithm. The first dimension is the parameter *k* of kNN, which can be set as a random integer within a certain range as required, but the value of k must be a positive integer. $s_{ij}\in \left[0,1\right]$, which is a random number, represents the j-th distance weight in the *i*-th solution. The evolutionary algorithm will continuously search and iterate under the guidance of the fitness function, and finally output the optimal solution or approximate optimal value [43], which is the most suitable kNN-related parameter. In order to demonstrate our model more clearly, the model’s structure is shown in Figure 2.

### 4.3. WSN Intrusion Detection System

The wireless sensor network (WSN) has become an increasingly important research area due to its wide range of real-time applications such as critical military surveillance, battlefields, building security surveillance, forest fire surveillance, and medical care [44]. A WSN consists of a large number of autonomous sensor nodes, which are distributed in different areas of interest to collect important data and wirelessly transmit the collected data to more powerful nodes, called sink nodes or base stations (BS) [45]. The data transmitted across the network depend on the dedicated WSN protocol, but the WSN is extremely vulnerable to attacks due to its open and distributed nature and the limited resources of sensor nodes.

**Figure 3:**
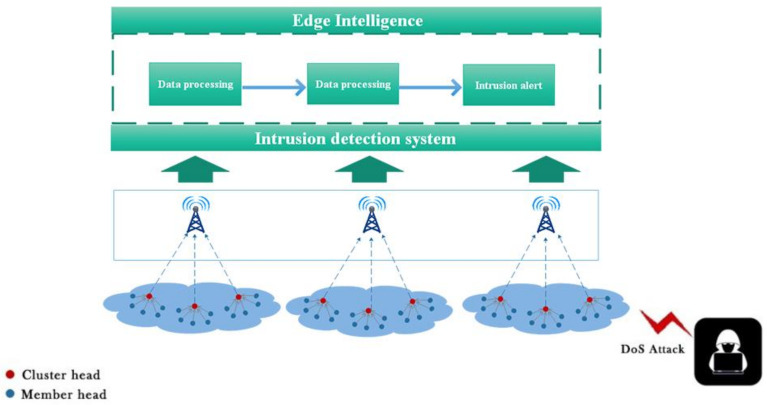
The model WSN intrusion detection system.

Since the process of avoiding or preventing security threats is not always successfully completed, an intrusion detection system (IDS) is needed to detect known and unknown attacks and send out alerts about them to sensor nodes [46]. An IDS can detect suspicious or abnormal activity and trigger an alarm when an intrusion occurs. The realization of a WSN IDS is more challenging than other systems, because sensor nodes are usually designed to be small and cheap, and they do not have enough hardware resources. In addition, there is no dedicated data set that contains general configuration files and attacks in WSN that can be used to detect attackers’ signatures. Considering the above challenges, we deploy edge nodes outside the WSN to enable the WSN to establish communication with the edge nodes, and we use edge intelligence to facilitate WSN intrusion detection. Figure 3 shows the edge-intelligent WSN intrusion detection system.

### 4.4. Performance Evaluation of Intrusion Detection System

Machine learning usually uses the following four criteria to evaluate the performance of a model: true positive (TP), true negative (TN), false positive (FP), and false negative (FN). They are also used to calculate various performance evaluation indicators, such as detection rate (DR), false positive rate (FPR), and accuracy rate (ACC). The calculation method is as follows:

(9) $$DR=TP/\left(TP+FN\right)$$

(10) $$FPR=FP/\left(FP+TN\right)$$

(11) $$ACC=\left(TP+TN\right)/\left(TP+FN+FP+TN\right)$$

Among them, *DR* represents the probability of a positive prediction in a sample with a normal actual value. *FPR* is the probability of a positive prediction in a sample with an abnormal actual value. *ACC* divides the number of samples correctly predicted by the total number of samples to indicate the accuracy of the prediction results. This article uses the *ACC* indicator as a fitness function, and its formula is as follows:

(12) $$fitness=\left(TP+TN\right)/\left(TP+FN+FP+TN\right)$$

## 5. Simulation Experiment and Analysis

### 5.1. The Experimental Results and Conclusions of PL-AOA

**Figure 4:**
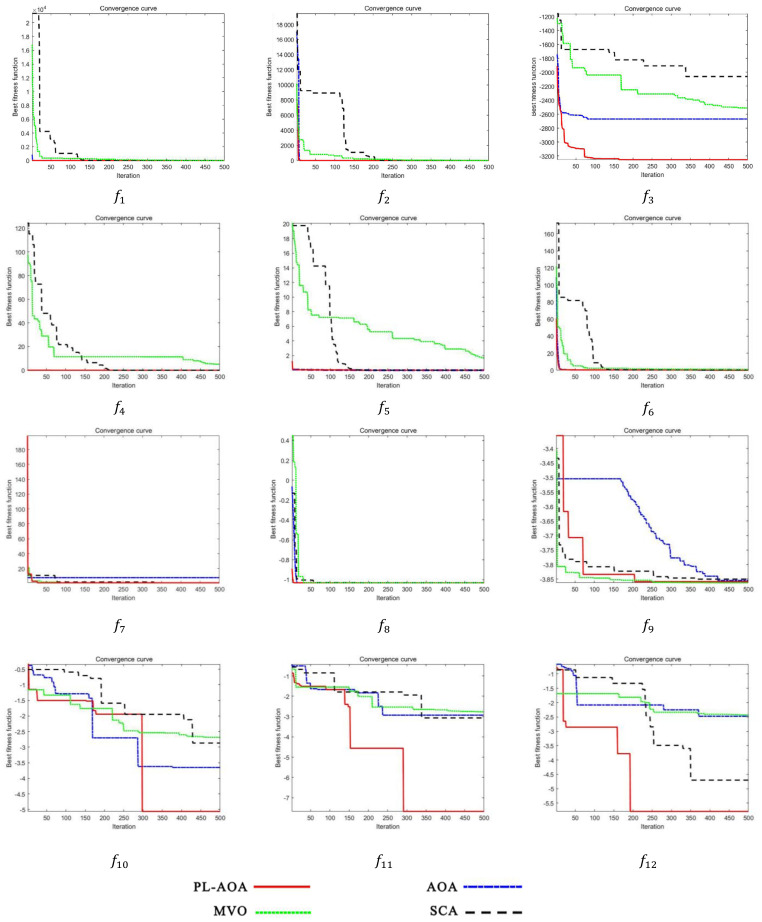
The convergence curves of test function.

As described in this section, in order to test the performance of PL-AOA, we used the original standard test function of AOA to conduct comparative experiments on PL-AOA, AOA, SCA [47], and MVO. This is a complex problem, so we designed the experiment as follows, focusing on testing the optimization of PL-AOA for complex functions. The test functions that we used included two single-mode functions ($f_{1}\sim f_{2}$), four multi-mode functions. ($f_{3}\sim f_{6}$), and six complex functions ($f_{7}\sim f_{12}$), as shown in Table 1. The test convergence curve is shown in Figure 4, and the test results are shown in Table 2.

Through three sets of benchmark function tests, it can be seen that PL-AOA has achieved the absolute advantage in algorithm comparison, and its performance in complex functions is more prominent. The numbers in bold indicate the best results for each set of tests which PL-AOA has achieved the nine best results in the 12 benchmark tests. In the standard deviation comparison, the first results were achieved eight times, which proves that the PL-AOA proposed in this paper has good optimization strength and reliable stability. Moreover, the average performance was improved by nearly 80% compared with the original AOA.

### 5.2. The Experimental Results and Conclusions of WSN Intrusion Detection System

In order to verify the practicability of the intrusion detection model proposed in this paper, the WSN intrusion detection data set WSN-DS [48] was used in simulation experiments. WSN-DS collects data from the network simulator 2 (NS-2), and then processes it to generate a data set of 23 features. Four types of DoS attacks are defined in WSN-DS: Blackhole, Grayhole, Flooding, and Scheduling attacks. The data distribution is shown in Table 3.

Before executing the algorithm, the data set was preprocessed, including numerical values, normalization, and other operations. The detection performance of four intrusion detection models (kNN, ${\mathrm{kNN}}_{\mathrm{PSO}}$, ${\mathrm{kNN}}_{\mathrm{AOA}}$, ${\mathrm{kNN}}_{\mathrm{PL}-\mathrm{AOA}}$) was tested. The experimental results are shown in Table 4, and the average results of 30 independent experiments were recorded. The total population of the three evolutionary algorithms of PSO, AOA, and PL-AOA was set to 20, and the number of iterations was 100.We can clearly find the model ${\mathrm{kNN}}_{\mathrm{PL}-\mathrm{AOA}}$ achieved the best results on the three indicators of ACC, DR, and FPR. This shows that the model can identify most of the DoS attacks that affect a WSN and can distinguish different types.

**Figure 5:**
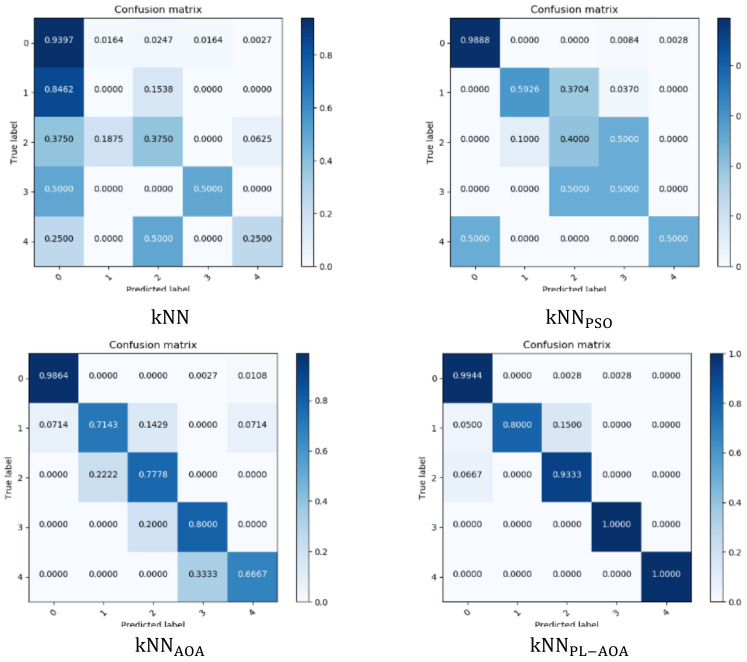
kNN, ${\mathrm{kNN}}_{\mathrm{PSO}}$, ${\mathrm{kNN}}_{\mathrm{AOA}}$, and ${\mathrm{kNN}}_{\mathrm{PL}-\mathrm{AOA}}$ confusion matrix.

In machine learning, the confusion matrix can be used to evaluate the accuracy of the four detection models. Here, we prove the accuracy of the four models by drawing the confusion matrix, as shown in Figure 5. The horizontal axis represents the predicted value, and the vertical axis represents the true value, visually showing the misclassification of each category. It can be seen that the ${\mathrm{kNN}}_{\mathrm{PL}-\mathrm{AOA}}$ model proposed in this paper has the best detection effect.

**Figure 6:**
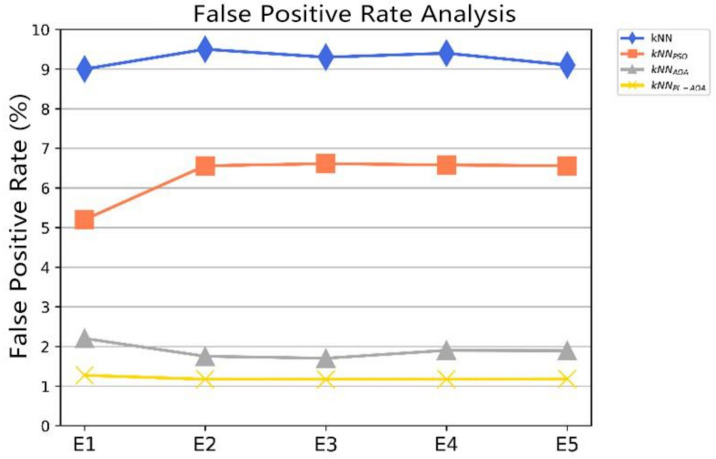
The false positive rate comparison for kNN, ${\mathrm{kNN}}_{\mathrm{PSO}}$, ${\mathrm{kNN}}_{\mathrm{AOA}}$, and ${\mathrm{kNN}}_{\mathrm{PL}-\mathrm{AOA}}$.

For WSN intrusion detection systems, reducing the false positive rate is a challenge. We conducted five independent experiments using the data set. Figure 6 visually shows the comparison results of the false positive rate of the four different detection algorithms. It can be seen that the false positive rate of ${\mathrm{kNN}}_{\mathrm{PL}-\mathrm{AOA}}\;$ is extremely stable at a low level.

**Figure 7:**
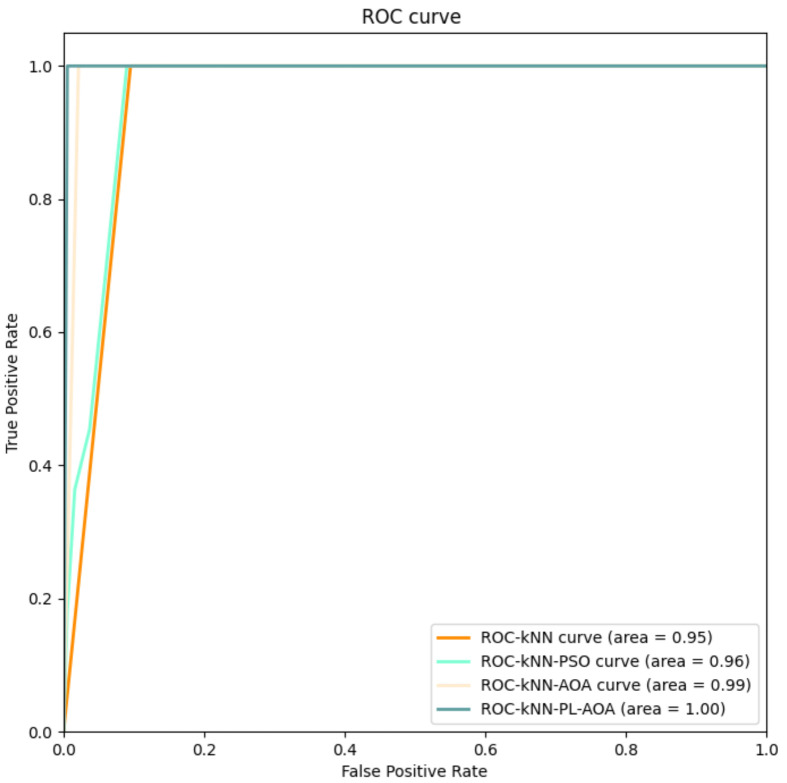
The AOC curves for kNN, ${\mathrm{kNN}}_{\mathrm{PSO}}$, ${\mathrm{kNN}}_{\mathrm{AOA}}$, and ${\mathrm{kNN}}_{\mathrm{PL}-\mathrm{AOA}}$.

Figure 7 shows the AOC curves of the four classification methods, where we can see that the ${\mathrm{kNN}}_{\mathrm{PL}-\mathrm{AOA}}$ proposed in the article achieves a good effect.

## 6. Conclusions

Due to the proposal of edge technology and its extensive combination with IoT devices, some complex technologies can be easily implemented in this way [49,50,51]. Some applications pose various security threats to WSNs, especially in unattended environments. In order to ensure the security and reliability of WSN services, an intrusion detection system (IDS) should be established. Intrusion detection is one of the key issues that urgently needs to be resolved in the practical application of WSNs. This paper proposes an edge-intelligent intrusion detection system that can be applied when a WSN encounters a DoS attack. First, we improve the AOA algorithm by using Lévy flight to improve the ability to jump out of the local optimum, and we use a parallel strategy to improve the population diversity in iterations. Then, through the combination of the improved PL-AOA optimization algorithm and kNN machine learning classifier, we not only improve the accuracy of detection and classification but also greatly improve the detection precision. The improved PL-AOA algorithm proposed in this paper has passed 12 benchmark function tests with outstanding results; we achieve the best results 9 times in 12 benchmark functions. In addition, the proposed intrusion detection model has been proven to be feasible in simulation experiments using WSN standard intrusion detection data sets to achieve a 99% ACC.

Time complexity is one of the evaluation indicators to measure the pros and cons of an algorithm. The time complexity of the PL-AOA proposed in this paper consists of two parts: initialization and solution update. The time complexity of the initialization process is $O\left(G\times N/G\right)=O\left(G\right)$. Due to the parallel strategy, regardless of how many groups are divided, the final population size is still N, G is the number of groups, and the solution update is $O\left(N\times T\right)+O\left(T\times N\times D\right)$, where T denotes the iteration and D denotes the dimension. The time complexity of Lévy flight is $\;O\left(1\right)$. Therefore, the time complexity of the PL-AOA algorithm is $O\left(N\times \left(TD+1\right)\right)$, as with the original AOA algorithm. The time complexity of kNN is $\;O\left(N\times D\right)$. In the proposed ${\mathrm{kNN}}_{\mathrm{PL}-\mathrm{AOA}}$, kNN is used as the update solution, so the time complexity of $kNN_{PL-AOA}$ is also $O\left(N\times \left(TD+1\right)\right)$. Although the time complexity is a little higher than that of the native kNN, the accuracy is improved by nearly 10%, so we believe that this time loss is an appropriate trade-off.

In the future, we will focus on developing an unsupervised or semi-supervised WSN intrusion detection model, such as k-means optimized by an evolutionary algorithm, and so on. These models will not only target a particular type of DoS attack, but also strive to cover Sybil attacks, routing attacks, and other possible attacks.

## Figures and Tables

**Table 1:** The 12 benchmark functions.

Function	Dim	Range	$F_{min}$
$f_{1}\left(x\right)=\sum_{i=1}^{n}\left|x_{i}\right|+\prod_{i=1}^{n}\left|x_{i}\right|$	30	$\left[-100,+100\right]$	0
$f_{2}\left(x\right)=\sum_{i=1}^{n}{\left(\sum_{j-1}^{i}x_{j}\right)}^{2}$	30	$\left[-100,+100\right]$	0
$f_{3}\left(x\right)=\sum_{i=1}^{n}-x_{i}\sin\left(\sqrt{\left|x_{i}\right|}\right)$	30	$\left[-1.28,+1.28\right]$	0
$f_{4}\left(x\right)=\sum_{i=1}^{n}\left[x_{i}^{2}-10\cos\left(2\pi x_{i}\right)+10\right]$	30	$\left[5.12,+5.12\right]$	0
$f_{5}\left(x\right)=-20\exp\left(-0.2\sqrt{\frac{1}{n}\;\sum_{i=1}^{n}x_{i}^{2}}\right)-\exp\left(\frac{1}{n}\sum_{i=1}^{n}\cos\left(2\pi x_{i}\right)\right)+20+e$	30	$\left[-32,+32\right]$	0
$f_{6}\left(x\right)=\frac{1}{4000}\sum_{i=1}^{\pi }x_{i}^{2}-\prod_{i=1}^{n}\cos\left(\frac{x_{i}}{\sqrt{i}}\right)+1$	30	$\left[-5.12,+5.12\right]$	0
$f_{7}\left(x\right)=\left(\frac{1}{500}*\sum_{i=1}^{25}\frac{1}{i+\sum_{i=1}^{2}\left(x_{j}-x_{ij}\right)}\right)$	30	$\left[-65,65\right]$	0
$f_{8}=4*x_{1}^{2}-2.1*\frac{x_{1}^{6}}{3+x_{1}*x_{2}}-4*x_{2}^{2}+4*x_{2}^{4}$	2	$\left[-5,+5\right]$	0
$f_{9}\left(x\right)=-\frac{1+\cos\left(12\sqrt{x_{1}^{2}+x_{2}^{2}}\right)}{0.5\left(x_{1}^{2}+x_{2}^{2}\right)+2}$	2	$\left[-2,+2\right]$	3
$f_{10}\left(x\right)=-\sum_{i=1}^{4}{\left[\left(X-a_{i}\right){\left(X-a_{i}\right)}^{T}+c_{i}\right]}^{-1}$	4	$\left[-10,+10\right]$	−10.1532
$f_{11}\left(x\right)=-\sum_{i=1}^{7}{\left[\left(X-a_{i}\right){\left(X-a_{i}\right)}^{T}+c_{i}\right]}^{-1}$	4	$\left[-10,+10\right]$	−10.4028
$f_{12}\left(x\right)=-\sum_{i=1}^{10}{\left[\left(X-a_{i}\right){\left(X-a_{i}\right)}^{T}+c_{i}\right]}^{-1}$	4	$\left[-10,+10\right]$	−10.5363

**Table 2:** Comparison results of PL-AOA, AOA, SCA, and MVO on 12 benchmark functions.

Function	Algorithm	Best Value	AVG	STD
$f_{1}$	PL-AOA	0	0	0
	AOA	0	0	0
	SCA	$2.09\times 10^{-14}$	$9.71085\times 10^{-9}$	$9.10029\times 10^{-9}$
	MVO	0.013127	0.0473636	0.025148762
$f_{2}$	PL-AOA	0	0	0
	AOA	$3.39\times 10^{-5}$	$6.7766\times 10^{-6}$	$1.35532\times 10^{-5}$
	SCA	$3.35\times 10^{-6}$	0.84790582	1.01309437
	MVO	0.23415	0.25472	0.167577958
$f_{3}$	PL-AOA	−3251.961	−3072.60658	192.0813616
	AOA	−2669.3232	−2828.61848	127.3300739
	SCA	−2060.5021	−2206.09518	222.1806306
	MVO	−2511.7046	−2889.61756	225.6564613
$f_{4}$	PL-AOA	0	0	0
	AOA	$1.85\times 10^{-8}$	$9.6634\times 10^{-14}$	$1.93268\times 10^{-13}$
	SCA	45.627	2.95231933	5.879472705
	MVO	9.9668	13.93912	4.664720195
$f_{5}$	PL-AOA	$8.88\times 10^{-16}$	$8.88\times 10^{-16}$	0
	AOA	$1.62\times 10^{-8}$	$3.25\times 10^{-9}$	$6.4912\times 10^{-9}$
	SCA	$9.33\times 10^{-8}$	$3.45\times 10^{-7}$	$2.14341\times 10^{-7}$
	MVO	0.040718	$5.09\times 10^{-2}$	0.016216161
$f_{6}$	PL-AOA	0	$2.14\times 10^{-12}$	$3.96128\times 10^{-13}$
	AOA	$2.34\times 10^{-14}$	$1.98\times 10^{-10}$	$8.2776\times 10^{-14}$
	SCA	0.0033891	$1.26\times 10^{-1}$	0.177027801
	MVO	0.20291	$3.81\times 10^{-1}$	0.134430398
$f_{7}$	PL-AOA	0.998	0.998	0
	AOA	7.874	2.377862	2.295234559
	SCA	0.99801	3.744742	3.61965409
	MVO	0.998	0.998	0
$f_{8}$	PL-AOA	−1.0316	−1.0316	0
	AOA	−1.0315	−1.03152	$9.79796\times 10^{-5}$
	SCA	−1.0314	−1.03124	0.000621611
	MVO	−1.0316	−1.02918	0.00459147
$f_{9}$	PL-AOA	−3.859	−3.85222	0.00484124
	AOA	−3.8549	−3.85486	0.003883607
	SCA	−3.8503	−3.85166	0.001654811
	MVO	−3.8628	−3.8628	0
$f_{10}$	PL-AOA	−5.0579	−5.04412	0.018240329
	AOA	−3.6494	−3.09772	0.96147737
	SCA	−2.8665	−2.53446	0.823849153
	MVO	−2.6828	−1.843174	1.282266985
$f_{11}$	PL-AOA	−7.6701	−7.3831	0.826224943
	AOA	−2.9294	−2.30354	1.040143612
	SCA	−3.0656	−2.12771	2.042497321
	MVO	−2.7659	−1.87218	3.060140019
$f_{12}$	PL-AOA	−5.7896	−4.91552	1.09829503
	AOA	−2.4736	−2.14244	1.972803802
	SCA	−4.699	−3.916174	1.515732845
	MVO	−2.4273	−1.36822	3.881249547
Compared with the four algorithms	Algorithm	Win	Win	Win
	PL-AOA	9	9	8
	AOA	0	0	1
	SCA	0	0	0
	MVO	1	1	1

**Table 3:** The data of WSN-DS.

Data Set	The Type of Data				
	Normal	Blackhole	Grayhole	Flooding	Scheduling Attacks
Number	340,066	10,049	14,596	3312	6638

**Table 4:** Classification effects of the four models.

Model	ACC (%)	DR (%)	FPR (%)
kNN	0.91162	0.95291	0.51429
${\mathrm{kNN}}_{\mathrm{PSO}}$	0.92893	0.94226	0.035714
${\mathrm{kNN}}_{\mathrm{AOA}}$	0.97727	0.97861	0.045455
${\mathrm{kNN}}_{\mathrm{PL}-\mathrm{AOA}}$	0.99721	0.99171	0.068966

## Data Availability

The data set is derived from ‘WSN-DS: A dataset for intrusion detection systems in wireless sensor networks’. https://doi.org/10.1155/2016/4731953 (accessed on 3 December 2021).
